# Effects of the tropical ginger compound,1’-acetoxychavicol acetate, against tumor promotion in K5.Stat3C transgenic mice

**DOI:** 10.1186/1756-9966-31-57

**Published:** 2012-06-15

**Authors:** Vinita Batra, Zanobia Syed, Jennifer N Gill, Malari A Coburn, Patrick Adegboyega, John DiGiovanni, J Michael Mathis, Runhua Shi, John L Clifford, Heather E Kleiner-Hancock

**Affiliations:** 1 Department of Pharmacology, Toxicology & Neuroscience, Louisiana State University Health Sciences Center, Shreveport, LA, USA; 2 Department of Biochemistry and Molecular Biology, Louisiana State University Health Sciences Center, Shreveport, LA, USA; 3 Department of Pathology, Louisiana State University Health Sciences Center, Shreveport, LA, USA; 4 Department of Cellular Biology & Anatomy, Louisiana State University Health Sciences Center, Shreveport, LA, USA; 5 Feist-Weiller Cancer Center, Shreveport, LA, USA; 6 School of Human Ecology, University of Texas, Austin, TX, USA; 7 Department of Medicine, Louisiana State University Health Sciences Center, Shreveport, LA, USA

**Keywords:** NF-κB, Stat3, Squamous cell carcinoma, Carcinogenesis, TPA, Tropical ginger

## Abstract

The purpose of the current study was to determine whether a tropical ginger derived compound 1’-acetoxychavicol acetate (ACA), suppresses skin tumor promotion in K5.Stat3C mice. In a two-week study in which wild-type (WT) and K5.Stat3C mice were co-treated with either vehicle, ACA, galanga extract, or fluocinolone acetonide (FA) and tetradecanoyl phorbol acetate (TPA), only the galanga extract and FA suppressed TPA-induced skin hyperproliferation and wet weight. None of these agents were effective at suppressing p-Tyr^705^Stat3 expression. However, ACA and FA showed promising inhibitory effects against skin tumorigenesis in K5.Stat3C mice. ACA also suppressed phospho-p65 NF-κB activation, suggesting a potential mechanism for its action.

## Introduction

An alarming rate of increase in the incidence of non-melanoma skin cancer (NMSC) is observed worldwide [1]. Within the United States itself, it has been estimated that about 1.7 million new cases of all forms of skin cancer are expected to be diagnosed each year [2]. To investigate the underlying pathophysiology of skin carcinogenesis, the multistage model delineates the cellular, biochemical and molecular processes involved in the various stages of skin cancer development [3-5]. Application of tumor promoters to initiated cells can induce epigenetic changes in the skin which culminate into visible clonal outgrowths known as papillomas [5-7]. Although the exact mechanism of action of tumor promotion remains unclear, sustained hyperplasia and cellular proliferation in the epidermis correlates with the tumor promoting activity. Moreover, treatment with tetradecanoyl phorbol acetate (TPA) can alter signaling of nuclear factor kappa B (NF-κB) and signal transducer and activator of transcription 3 (Stat3) signaling in the process of skin carcinogenesis [8]. Stat3 is a transcription factor that plays a critical role in the control of cell proliferation, survival and angiogenesis, all hallmarks of malignancy [9]. Stat3 activity is constitutive in several malignant cell types and is required for initiation, promotion and progression to a more malignant phenotype in squamous cell carcinomas of the skin (SCC) [8,10-15]. The critical role of Stat3 in skin tumor development was further supported by data obtained from the K5.Stat3C transgenic mouse model in which the DiGiovanni and Clifford research groups expressed the Stat3C protein in skin under the control of the keratin-5 promoter [11]. Stat3C is a constitutively active mutant of Stat3 that dimerizes through formation of covalent disulfide linkages between cysteines instead of phosphotyrosines [16]. These mice have a skin phenotype closely resembling psoriasis in humans and, when subjected to the two-stage skin chemical carcinogenesis protocol, rapidly developed carcinomas, bypassing the papilloma stage that is normally observed in this model [17].

The transcription factor NF-κB is also activated during inflammation and carcinogenesis [18]. The activated form of NF-κB triggers transcription of specific genes involved in proliferation (cyclin D1, c-myc), angiogenesis (VEGF), antiapoptosis (survivin, BclXL, FLIP) and invasion (MMP9, ICAM-1) proteins [19]. NF-κB activation has been strongly implicated in many types of cancer [18] including skin SCCs [20]. Ablation of β-catenin in murine skin grafts resulted in up-regulation of NF-κB target genes [21]. The skin grafts, which resembled human grade III skin SCCs, were hyperproliferative, the layers of epidermis were disorganized, and contained invasive keratinocytes [21]. Kobielak and Fuchs analyzed human skin SCCs and found 33/40 with low/no β-catenin, and nuclear, activated NF-κB, also characterized by inflammation and interestingly, nuclear phosphorylated Stat3 [21]. Finally, many NF-κB regulated genes are also induced by Stat3 and the interaction between these proteins and their signaling pathways may be involved in the different phases of skin carcinogenesis.

Non-specific drug-related side effects of pharmaceuticals hamper their clinical efficacy and underscore the need for investigating better treatment options. Cruciferous vegetables, tomatoes, garlic, citrus fruits and beverages like black tea and green tea contain phytochemicals such as resveratrol, flavonoids, and lycopene, that have been shown to afford protection against skin cancer development in vivo [22-24]. Easy accessibility and cost-effectiveness provide a reasonable rationale to explore phytochemicals for mechanism-based interventions in cancer management. ACA is a natural component of traditional Thai condiments found in the seeds, rhizomes or in the root of the tropical ginger [25]. ACA suppressed carcinogenesis in a number of rodent models, including the two-stage mouse skin model [26,27], the 4-nitroquinoline oxide oral carcinogenesis model [28,29], and the azoxymethane colon carcinogenesis model [30,31]. In the skin model, pre-treatment of mice with ACA during TPA treatment in 7, 12-dimethylbenz [a] anthracene (DMBA)-initiated mice was remarkably effective, inhibiting skin tumor promotion by 44 % and 90% at 1.6 nmol and 160 nmol doses, respectively [27]. Some of the proposed anticarcinogenic mechanisms of ACA included the ability to inhibit ornithine decarboxylase (ODC) activity, inhibition of xanthine oxidase and suppression of the formation of superoxide anion, induction of detoxifying enzymes, and causing apoptosis in cancer cells [29,30,32-35]. We found that ACA induced apoptosis in human breast carcinoma MDA-MB-231 cells [36]. ACA was also shown to inhibit the formation of reactive oxygen species by suppressing leukocyte infiltration in the dermis following TPA exposure [35]. It was also found that ACA blocked TNFα induced activation of NF-κB indirectly through IκB [37].

Because of the strong role of Stat3 and NF-kB in SCC, and the dramatic effect of ACA against skin tumor promotion, we hypothesized that the effects of ACA may be modulated through Stat3 and/or NF-κB signaling. To address this question, we used mice that express the constitutively active form of Stat3 (K5.Stat3C). Moreover, ACA exists in nature exclusively as the *S*-enantiomer, while the synthetic form utilized in most experimental studies is the racemic mixture. In order to determine whether there are differences in biological effects between the ACA-S and the racemic mixture, we tested ACA-S in the form of a galanga extract (hereafter referred to as GE), alongside synthetic ACA.

## Materials and methods

### Preparation of dosages

Synthetic 1’-acetoxychavicol acetate (ACA) was purchased from LKT Laboratories (St. Paul, MN). Fluocinolone acetonide (FA) was purchased from Sigma-Aldrich (St. Louis, MO). Tetradecanoyl phorbol acetate (TPA) was purchased from LC Laboratories (Woburn, MA). All solutions of ACA, FA and TPA were prepared in HPLC grade acetone and were applied topically in a total volume of 0.2 mL. The dose of TPA used in the subsequent experiments was 3.4 nmol. Based on our previous dose–response studies [38], 340 nmol of ACA was used for all the experiments presented. The dose of FA used was 2.2 nmol in 0.2 mL per mouse.

### Preparation of galangal extract

The rhizome of *Alpinia galanga* was obtained from a local market in Shreveport, LA. Two separate extracts were made: one in ethanol and the other in hexane. All procedures were conducted in subdued lighting. 100 g of fresh rhizome was chopped into small pieces and mixed with either 500 mL of HPLC grade 100% ethanol or hexane. This extract was stored for a week, protected from light, at 4 °C followed by daily shaking the flask in order to allow the contents to mix well. The extract was analyzed by HPLC-UV detection (Shimadzu Scientific Instrument, Columbia, MD) on an ODS-3 5 μ column at 1 mL/min in 70% methanol/water at 254 and 213 nm. There was a 1000-fold difference observed in the areas under the curve (AUC) for ACA at 254 and 213 nm wavelengths with the AUC being greater at 213 nm. A peak corresponding to the authentic standard ACA eluted at 9.1 min. The retention time of the predominant peak in the galanga extract was compared to that of synthetic ACA and they were found to be the same. The concentration of ACA was found to be 3.8 mM in the ethanolic extract and 2 mM in the hexane extract. Both extracts possessed numerous other peaks yet to be identified. Interestingly, there were several peaks identified in the ethanolic extract that were not observed in the hexane extract. The ethanolic extract also possessed a more fragrant aroma that developed over time. Both extracts developed an amber color over time. Because the ethanolic extract was difficult to dry down, the hexane derived extract was used for experiments. The hexane extract was dried under nitrogen gas to make a concentrate that was further resuspended in HPLC grade acetone, analyzed by HPLC against an authentic standard curve, and diluted such that 340 nmol of ACA per 0.2 mL was obtained.

### Cell culture

The Clifford laboratory generated several clones of SENCAR mouse keratinocyte-derived cells (3PC) stably expressing the Stat3C protein (3PC-C1, 3PC-C10, 3PC-C17, etc.). Overexpression of Stat3C sensitized these cells to EGF and HGF induced cell migration, and invasion through Matrigel [17]. 3PC parental cells (3PC WT) and 3PC-C10 cells were grown in chelexed EMEM media (0.05 mM Ca^2+^, 5 ng/ml epidermal growth factor, 10 μM ethanolamine, 4 mM glutamine, 1 μM hydrocortisone, 5 μg/ml insulin, 100 μg/ml penn-strep, 10 μM phosphoethanolamine and 10 μg/ml transferrin) supplemented with 8% chelexed FCS, in a humidified atmosphere with a 5% CO_2_ concentration. Cells were seeded onto 96-well plates and treated with vehicle (0.1% DMSO) or ACA (2.5, 5, and 10 μM) for 96 h. Plates were harvested for the MTT viability assay as previously described [13].

### General animal care

All animals were kept in a temperature and humidity controlled AAALAC facility under a normal 12 hour light/dark cycle. The procedures were approved by LSUHSC Institutional Animal Care and Use Committee in accordance with NIH guidelines. Mice were maintained on regular pellet food and allowed access to food and water *ad libitum.* Transgenic mice with constitutive Stat3 expression (K5.Stat3C) and their wild-type (WT) counter parts were used. K5.Stat3C mice express Stat3C, a constitutively active form of Stat3 and develop spontaneous lesions that resemble human psoriasis [11]. The expression of the Stat3C transgene in the basal cell layer of the epidermis was driven by the bovine keratin 5 gene promoter, and hence the name K5.Stat3C. The mice were genotyped by PCR to detect the transgene and maintained in the breeding colony at LSUHSC-Shreveport.

### Effects of ACA, galanga extract, and FA on mouse epidermis following two weeks treatment with TPA in WT vs. K5.Stat3C mice

The dorsal skin of each mouse was shaved two days prior to the treatments. At 2 days post shaving, topical applications of respective treatments were administered on the dorsal surface of the mouse with the aid of a pipette, according to the two-week protocol reported previously for short-term tumor promoter experiments [8]. The mice were treated twice weekly for two weeks as follows; treatment with either acetone vehicle, synthetic ACA (340 nmol), galanga extract (equivalent of 340 nmol ACA) or FA (2.2 nmol), followed by treatment with TPA (3.4 nmol). Mice were sacrificed 48 h after the last treatment application and tissues were harvested for further experimental analysis. The dorsal skin from the mice was excised and divided into three parts; one for wet weight analysis, one for histological analysis, and one for western blot analysis. For wet weight analysis, the underlying fat layer was dissected from one of the skin pieces and two holes were punched into the excised skin, one towards the rostral end and the other towards the caudal end. The punched biopsies were then placed into vials and weighed on an analytical balance (AG135, Mettler-Toledo, Inc., Columbus, OH). The weights of the biopsies obtained from the rostral and caudal end were then averaged for each individual mouse and recorded.

For histological analysis, one piece of skin was placed in 10% neutral buffered formalin, and at 24 hrs post fixation transferred into 50% ethanol and embedded in paraffin. The tissue sections were sliced crossectionally at a thickness of 4 μm. Duplicate histology sections were stained with hemotoxylin and eosin for histopathological analysis. Epidermal thickness was measured using the hematoxylin and eosin stained histology slides. Digital images of the histology slides were captured using a Nikon Eclipse TE300 inverted microscope with an epi-fluorescence attachment. This was attached to Photometrics CoolSNAP_fx_ monochrome 12-bit CCD camera and configured with imaging Software: IPLab 3.7 for Windows (Research Core Facility, LSUHSC). The procedure for measuring epidermal thickness reported by Li, Wheeler and colleagues was followed with slight modifications [39]. Digital pictures of 10 randomly selected fields were taken at 400X magnification. The sections were scored in a blinded fashion such that the slides only had a numerical identity. For each skin site, epidermal thickness was measured vertically from the basal layer up to, but excluding, the stratum corneum using “Metamorph Software” (Research Core Facility, LSUHSC). Image distances were calibrated using a hemocytometer grid photographed on the same microscope and at the same magnification as the histology images, allowing a pixel to microns conversion factor to be obtained at 400X magnification. One pixel was equal to 0.16722 μm. For each individual mouse, twenty measurements were recorded and the values averaged for analysis.

For western blot analysis, excised skin was placed on a glass plate on ice followed by removal of the epidermis with a razor blade. The epidermal scrapings were placed into RIPA lysis buffer (50 mM Tris–HCl, pH7.4, 1% NP-40, 150 mM NaCl, 1 mM EDTA, 1 mM PMSF, 1ug/mL leupeptin, 1ug/mL aprotinin, 1 mM Na_3_VO_4_, 1 mM NaF [Abcam, Cambridge, MA], and 1X protease inhibitor cocktail [Sigma-Aldrich, St. Louis, MO]), and homogenized on ice using a polytron homogenizer with 3 bursts of 30 sec each, followed by intermittent resting 10 sec between each burst and then centrifuged at 14,000 x g for 15 min at 4 °C. The supernatant (epidermal lysate) was collected, quantitated using Bio-Rad Protein Dye and according to the method of Bradford as previously described [40], and used for Western blot analysis. Epidermal lysates were separated by SDS-PAGE, electrophoretically transferred to a PVDF membrane, followed by staining with Ponceau S to assure efficient transfer. The blots were probed with antibodies for Stat3 and P^Tyr705^Stat3 (Cell Signaling Technology, Inc., Beverly, MA) and signal intensity quantitated as previously described [15].

### Tumor study

K5.Stat3C (male and female) mice (6–8 weeks of age) were initiated with 25 nmol DMBA and then treated with TPA (6.8 nmol) twice a week for the duration of the study as previously described [17]. Mice were pre-treated with 340 nmol ACA or 2.2 nmol FA 5 min prior to each TPA treatment. Mice were palpated for tumors twice weekly for the duration of the study. The numbers of subjects in each group were 14 (TPA only), 10 (ACA/TPA) and 6 (FA/TPA). At the end of the study, mice were euthanized, and skin and tumors were removed for histopathological analyses and immunohistochemistry (IHC).

### Statistical analysis

Statistical analysis was performed using GraphPad Prism ^R^ version 3.0 software for Windows (GraphPad Software, San Diego, CA). The statistical analysis used for these studies was One way ANOVA followed by Tukey’s Multiple Comparison Test as the post test, with p < 0.05 being the level of significance. For the tumor study, multiplicity was analyzed using the Kruskal-Wallis non-parametric test (GraphPad Prism ^R^ version 5.0 for Mac).

## Results

### Effects of ACA on cells that overexpress Stat3

**Figure 1:**
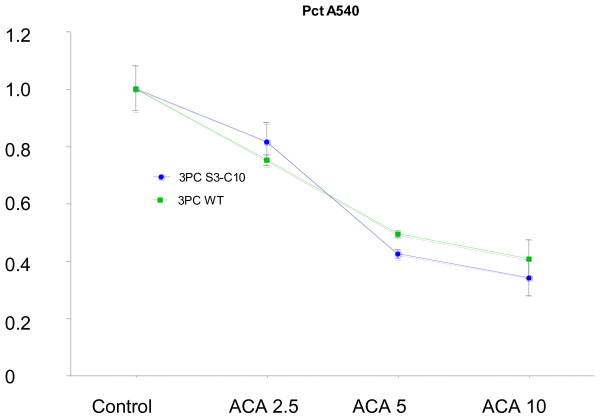
**Effects of ACA in 3PC and 3PC-C10 cells.** Cells were cultured as described in Methods sections and cell viability and/or proliferation was assayed by the MTT method. Figures represent triplicate values. The experiment was repeated with similar results. Data are expressed as the percentage of the vehicle control (y-axis, ratio of experimental group to control group).

In order to determine whether these cells were sensitive to the antiproliferative and/or cell killing effects of ACA, a dose response viability assay was performed. The absolute absorbance value at 540 nm for the 3PC-C10 cells was higher than that of the 3PC control cells. ACA significantly suppressed MTT color development by ~ 20% - 60% (2.5 – 10 μM) (Figure 1). A linear trend analysis demonstrated that there was a significant decrease of absorbance at 540 nm with increase of dose for both cell lines. However, when the data were expressed as a percentage of control (Figure 1), there was no interaction effect between cell type and treatment, suggesting that the cells are equally sensitive to ACA.

### Effects of ACA, galanga extract, and FA on mouse epidermis following two weeks treatment with TPA in WT vs. K5.Stat3C mice

**Figure 2:**
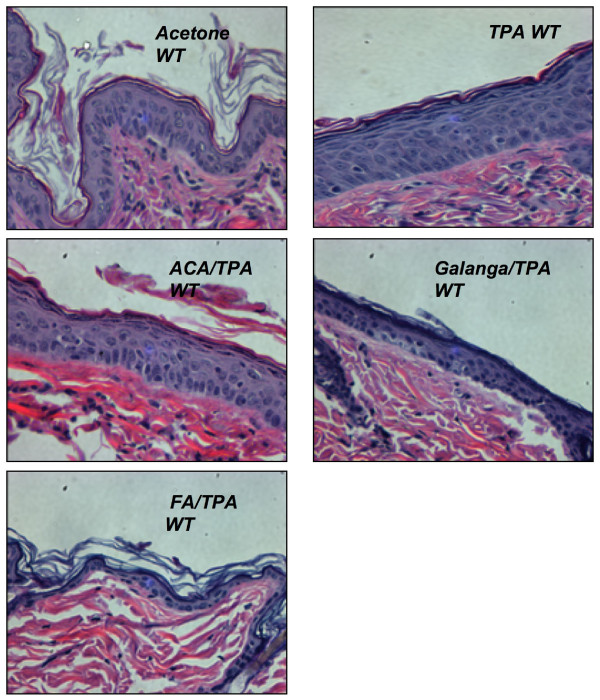
**Effect of ACA, galanga extract, and FA in TPA-treated WT mouse skin.** Wild-type (WT) mice were treated with TPA ± ACA, galanga extract, or FA twice a week for 2 weeks. H&E photomicrographs at 400X. Males and females (n = 6-8) were used. Treatment groups were vehicle/vehicle; vehicle/TPA 3.4 nmol; ACA 340 nmol/TPA 3.4 nmol; galanga extract (GE, equivalent to 340 nmol ACA)/TPA 3.4 nmol and FA 2.2 nmol/TPA 3.4 nmol.

**Figure 3:**
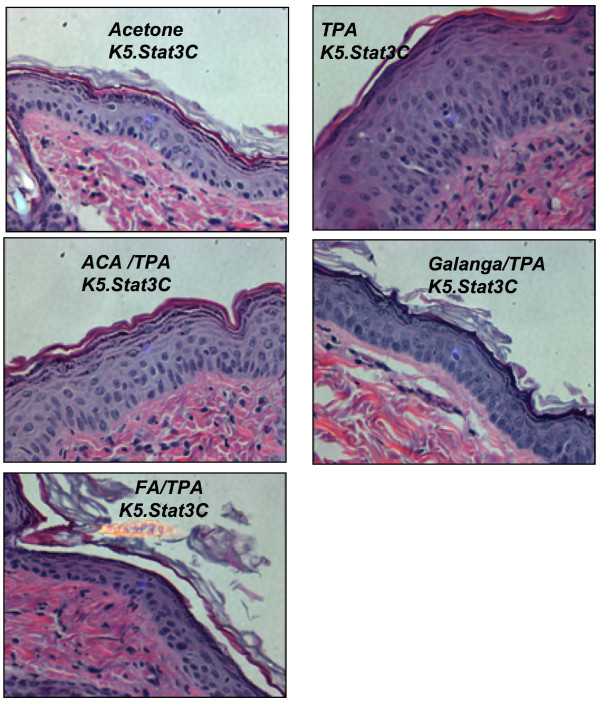
**Effect of ACA, galanga extract, and FA in TPA-treated K5.Stat3C mice mouse skin.** K5.Stat3C mice were treated with TPA ± ACA, galanga extract, or FA twice a week for 2 weeks. H&E photomicrographs at 400X. Males and females (n = 6-8) were used. Treatment groups were vehicle/vehicle; vehicle/TPA 3.4 nmol; ACA 340 nmol/TPA 3.4 nmol; galanga extract (GE, equivalent to 340 nmol ACA)/TPA 3.4 nmol and FA 2.2 nmol/TPA 3.4 nmol.

**Figure 4:**
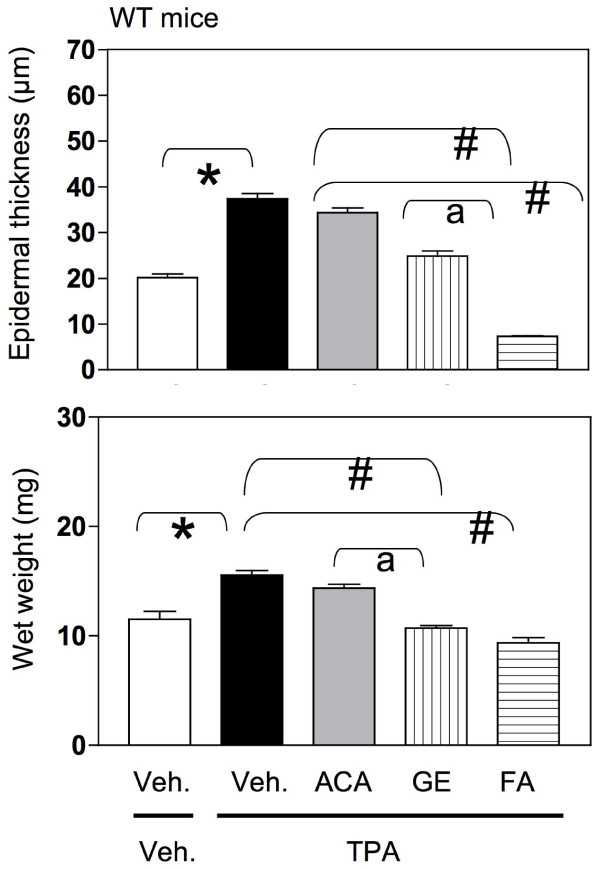
**Effect of ACA, galanga extract, and FA on epidermal thickness (top panels) wet weight (lower panels) in TPA-treated WT mouse skin.** WT mice were treated with vehicle/vehicle; vehicle/TPA 3.4 nmol; ACA 340 nmol/TPA 3.4 nmol; galanga extract (GE, equivalent to 340 nmol ACA)/TPA 3.4 nmol and FA 2.2 nmol/TPA 3.4 nmol twice a week for 2 weeks.

**Figure 5:**
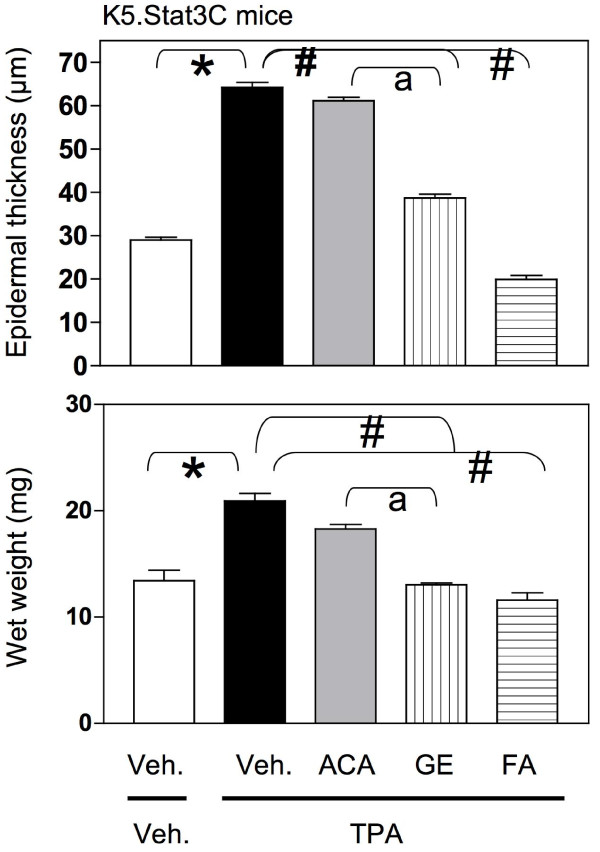
**Effect of ACA, galanga extract, and FA on epidermal thickness (top panels) wet weight (lower panels) in TPA-treated K5.Stat3C mouse skin.** K5.Stat3C mice were treated with vehicle/vehicle; vehicle/TPA 3.4 nmol; ACA 340 nmol/TPA 3.4 nmol; galanga extract (GE, equivalent to 340 nmol ACA)/TPA 3.4 nmol and FA 2.2 nmol/TPA 3.4 nmol twice a week for 2 weeks.

**Figure 6:**
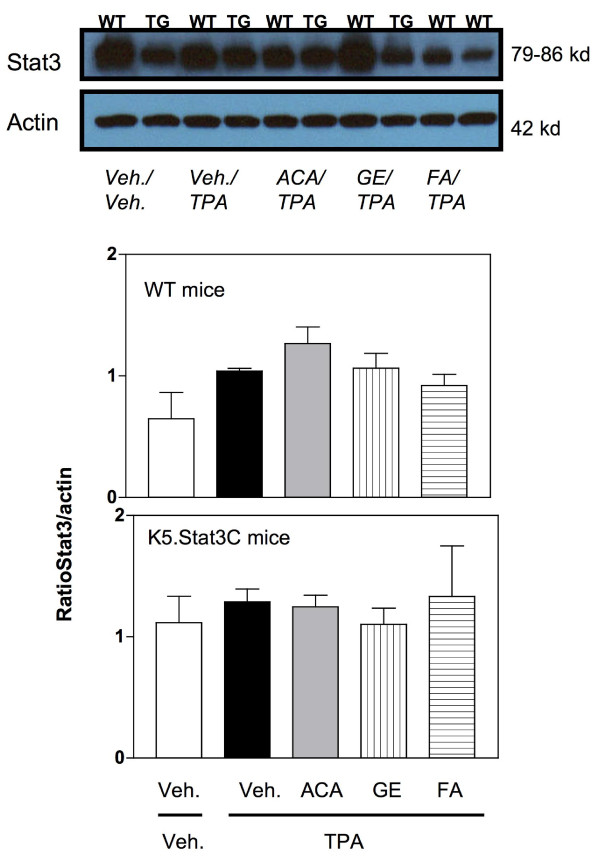
**Western blot analysis of to Stat3 expression in wild-type (WT) mouse epidermis.** TPA (3.4 nmol) was administered twice a week for 2 wk and mice were euthanized at 48 h. Mice were co-treated with vehicle (acetone 200 μL), ACA (340 nmol), galanga extract (GE, corresponding to 340 nmol ACA) or FA (2.2 nmol). Figures represent densitometry analysis of ratio of Stat3/actin (panel A); and p-Tyr^705^Stat3/actin panel B (Means ± SE of 6–8 individual mice).

**Figure 7:**
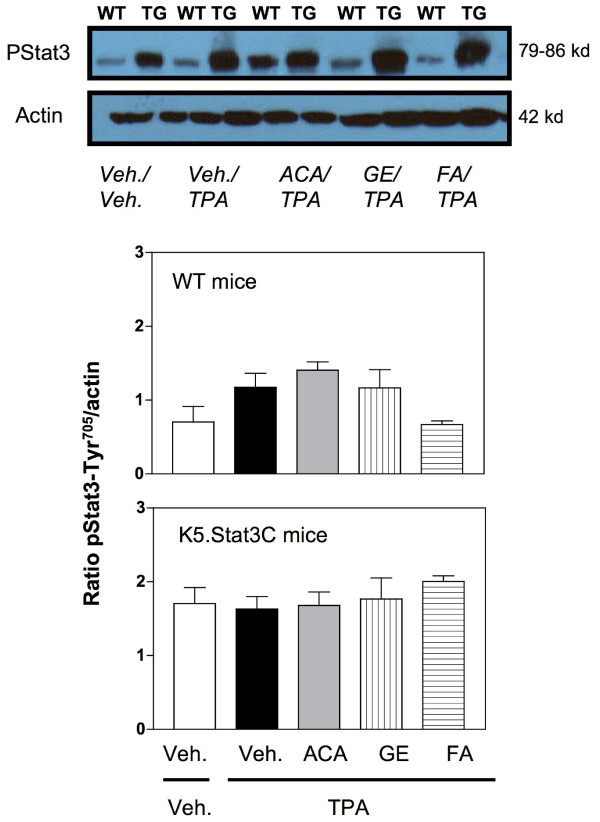
**Western blot analysis of to Stat3 expression in K5.Stat3C transgenic (TG) mouse epidermis.** TPA (3.4 nmol) was administered twice a week for 2 wk and mice were euthanized at 48 h. Mice were co-treated with vehicle (acetone 200 μL), ACA (340 nmol), galanga extract (GE, corresponding to 340 nmol ACA) or FA (2.2 nmol). Figures represent densitometry analysis of ratio of Stat3/actin (panel A); and p-Tyr^705^Stat3/actin panel B (Means ± SE of 6–8 individual mice).

To understand the histological changes in the epidermal layer of the subjects under the influence of various treatments, hematoxylin and eosin staining was done. Figures 2, 3 show a representative image of the histology sections from the various treatment groups. These histological differences were further quantified as epidermal thickness and are reported in Figure 4, Figure 5, Figure 6 and Figure 7.

In the WT mice, the epidermis in the vehicle/vehicle group was only a few layers thick when observed from the basal layer up to the stratum corneum (Figure 2) and the nucleated cells in the basal layer appeared to be round and light in color. The thickness of the epidermis in this group was approximately 18–21 μm (Figure 4, top panel). On the other hand, the epidermis in the vehicle/TPA group was several cell layers thicker (Figure 2). The quantitative result showed a marked elevation in the thickness and was about 38 μm when compared to the vehicle control (Figure 5, top panel). The epidermis in the synthetic ACA/TPA treated group resembled the TPA treated epidermis with no significant changes in the thickness (Figures 2 and 4). However, the epidermis in the galanga extract/TPA treated group looked very similar to the acetone control group with only only a few layers thick and quantitatively measured to be approximately 25 μm (Figures 2 and 4). The thickness in this group was significantly less in comparison to TPA treated group. The epidermal thickness in the galanga extract treated group was significantly lower in comparison to the ACA treated group. Interestingly, as previously reported, FA treated subjects had a very thin, atrophic epidermis which was to be around 6–7 μm (Figures 2 and 4). The thickness of the epidermis in this group was significantly reduced by about 3-fold in comparison to the TPA treated group.

In the K5.Stat3C mice, (Figures 3 and 5) similar results were observed across all the treatment groups as seen with the non-transgenic mice with the only differences noticed in the basal levels of the epidermal thickness in the transgenic mice and their non-transgenic littermates. This difference in the basal levels of the epidermal thickness was mainly observed due to the phenotypic differences in the skin of the transgenic mice and their WT counterparts. These results suggested that galanga extract as well as FA were effective agents in modulating the cellular events associated with the promotional phase of skin cancer.

Changes in wet weight also corresponded to changes in epidermal thickness (Figure 4, lower panel). The effect of various treatments on wet weight was also assessed. Wet weight is an indicator of edema as well as hyperproliferation, both markers of skin tumor promotion induced by TPA [41]. In Figure 4, lower panel, the wet weight of the WT skin in the vehicle only group was 10–13 mg whereas the wet weight in vehicle/TPA group comparatively was significantly increased to 14–16 mg. The wet weight in the group treated with synthetic ACA/TPA was similar to the vehicle/TPA treated group without any significant changes in the wet weight of the skin. However, the wet weight of skin in the group treated with galanga extract/TPA was significantly decreased in comparison to the vehicle/TPA treated group. Furthermore, the wet weight of the skin in the FA/TPA treated group was also significantly reduced in comparison to the vehicle/TPA treated group. Interestingly, the wet weight in the galanga extract/TPA group was significantly lower than the wet weight in the synthetic ACA/TPA treated group.

In Figure 5, lower panel, the wet weight in the vehicle only K5.Stat3C group was 14–15 mg, which was slightly higher than the wet weight observed in the WT group. In the vehicle/TPA treated K5.Stat3C group, the wet weight was significantly higher when compared to the vehicle only group. Yet again, the basal level of wet weight in this group was slightly higher in comparison to the WT group. The difference in the basal levels of the wet weight in the transgenic mice and their non-transgenic littermates were observed across all the treatment groups. In comparison to the vehicle/TPA group, the wet weight was significantly lower in the galanga extract/TPA and FA/TPA treated groups but not in the synthetic ACA/TPA group. Moreover, the wet weight of skin in the galanga extract/TPA group was significantly lower in comparison to synthetic ACA/TPA treated group. This suggested that the test agents gave similar results in the transgenic mice and their non-transgenic littermates, with the galanga extract being more effective than synthetic ACA. FA was once again found to be effective in decreasing the wet weight of the skin.

To address the effects of the various treatments on the potential molecular target, Stat3, semiquantitative Western blot analysis for the expression of Stat3 and its active form (i.e. phosphorylated form of Stat3 at tyrosine residue 705) was performed. Figure 6 shows a representative western blot for Stat3 expression. As per our expectations, the expression of Stat3 remained unchanged in all the WT treatment groups (Figure 6, middle panel). This was a consistent observation reported by several other researchers in the literature [8,42]. Further, Figure 6, lower panel, shows the experimental data for Stat3 expression in the K5.Stat3C mice. Once again, there were no significant differences observed in the expression of the Stat3 protein itself by any of the treatments.

However, modulation of phosphorylated Stat3 expression as a function of the particular agent treatment was observed. Figure 7, top panel, shows a representative Western blot for the active form of Stat3 expression, i.e. phosphorylated Stat3 at tyrosine residue 705. In Figure 7, middle panel, the experimental data for the phosphorylated Stat3 expression in WT mice are shown. As evident from the data presented, TPA treatment did not significantly increase the expression of phosphorylated Stat3 in comparison to the vehicle control. It could be that activation of Stat3 occurred earlier than 48 h. Moreover, neither the synthetic ACA nor the galanga extract was effective in modulating the expression of phosphorylated Stat3. The effect of FA was not significantly different from the TPA treated group. In Figure 7, lower panel, data for the K5.Stat3C transgenic mice only are shown. An important point to be considered is that these mice have constitutive expression of Stat3 in the epidermal keratinocytes which also means these mice have the active Stat3 or phosphorylated Stat3 signal already turned on. Therefore, these mice have higher basal levels of the phosphorylated Stat3 protein as compared to the basal levels of this protein in the wild type mice. Once again, TPA did not increase the expression of phosphorylated Stat3 in the transgenic mice. Furthermore, neither synthetic ACA nor the galanga extract was able to modulate the expression of the phosphorylated Stat3 protein in the transgenic mice. Even FA was not able to shut off the activated Stat3 signal in the transgenic mice and thus did not modulate the expression of phosphorylated Stat3 as it did in the wild type mice previously.

### Effects of ACA and FA on skin carcinogenesis in WT vs. K5.Stat3C mice

**Figure 8:**
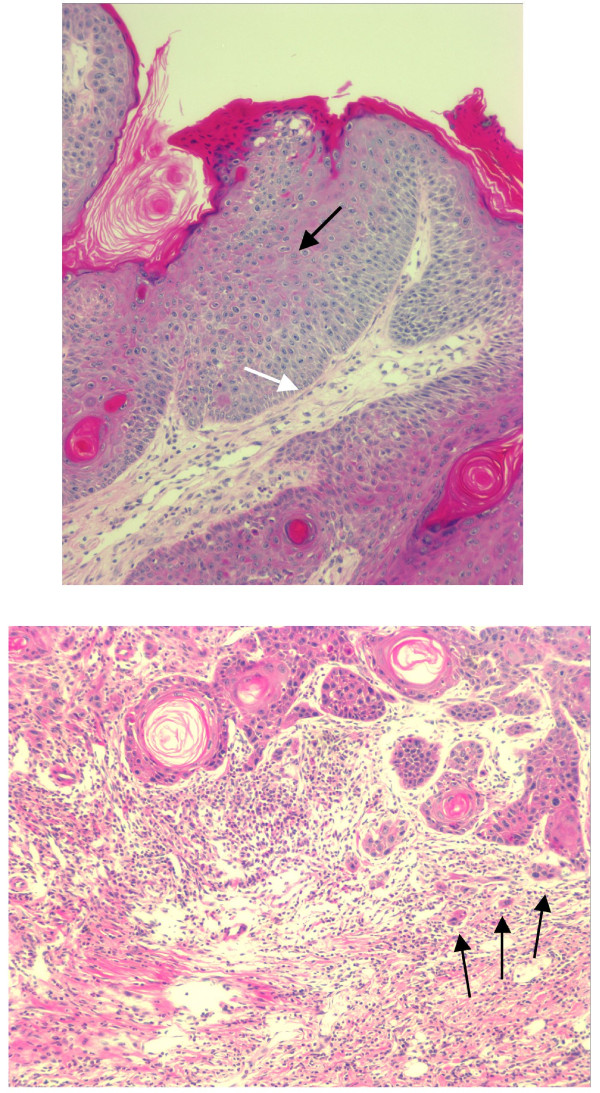
**Representative H&E photomicrographs of carcinoma in-situ (top panel) and invasive SCC (lower panel).** Top panel, markedly thickened epithelial layer with multiple layers of cells and dysplasia (nuclear atypia, black arrow). White arrow points to the rounded outline without breaching the basement membrane, denoting the pre-invasive phase (ie., carcinoma in-situ). Lower panel, micrograph showing irregular nests (black arrows) of proliferating epithelial cells with cellular atypia and nuclear polymorphism. The tumor nests (black arrows) are seen infiltrating into the stroma as single cells and irregular nests (black arrows) (original magnification 200x).

Finally, the effects of ACA on DMBA/TPA-induced tumorigenesis were examined in K5.Stat3C transgenic mice (Tables 1–2, Figure 8). In the K5.Stat3C mice treated with TPA only, lesions began to appear between 5–16 weeks of promotion and reached a maximum at 21 weeks. This experiment was terminated at 21 weeks due to morbidity in the TPA only mice. Statistical analyses of the histopathology are summarized in Tables 1–2. Overall, there were fewer carcinomas in-situ than invasive SCCs (Table 2). The percentages of mice with carcinomas in-situ were not statistically significant (Table 1). However, the percentages of mice with invasive SCC’s were significantly different, with the FA/TPA group being significant and the ACA/TPA group being marginal, suggesting that more subjects in the ACA/TPA group might have revealed a difference. Histopathological analyses revealed an average of 1.21 ± 0.38 carcinomas in-situ and 3.07 ± 0.61 invasive SCC’s per mouse in the TPA only group (Table 2). There was no significant difference in the average numbers of carcinomas in-situ. However, there was a significant difference in the average numbers of invasive SCC with the FA/TPA group being significant and the ACA/TPA group being marginal, again suggesting that more subjects in the ACA/TPA group might have revealed a difference. There were no significant differences between the ACA/TPA group and the FA/TPA group in either incidence or multiplicity (statistics not shown).

**Table 1:** Histopathological Analyses of Tumor Incidence

**Treatment**	**% of Mice with Carcinoma in-Situ^a^**	
TPA	57.1%	
TPA/ACA	33.3%	
TPA/FA	33.3%	
Exact p-value	0.4942	
	**% of Mice with Invasive SCC**^**a**^	
TPA	100%	Compared to TPA^b^
TPA/ACA	72.7%	p = 0.0717
TPA/FA	33.3%	p = 0.0031
Exact p-value	0.0031	

^a^ SAS System, Pearson Chi-Square Test.

^b^ Fisher's Exact Test.

**Table 2:** Histopathological Analyses of Tumor Multiplicity

**Treatment**	**Avg no. of Carcinomas in-Situ^d^**	
TPA	1.21 ± 0.38	
TPA/ACA	0.44 ± 0.24	
TPA/FA	0.33 ± 0.21	
LS-Means ^e^	P = 0.1592	
	**Avg no. of Invasive SCC**^**d**^	
TPA	3.07 ± 0.61	Compared to TPA^f^
TPA/ACA	1.54 ± 0.34	p = 0.1164
TPA/FA	0.83 ± 0.65	p = 0.0476
LS-Means ^e^	P = 0.0324	

^d^ Means ± SE.

^e^ SAS System, GLM Procedure, Least Squares Means Test.

^f^ Adjustment for Multiple Comparisons: Tukey-Kramer.

**Figure 9:**
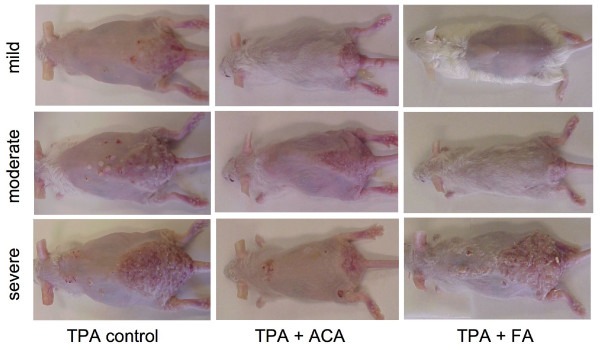
**Representative photographs taken of mice from each group exhibiting mild, moderate, and severe psoriatic phenotypes.** K5.Stat3C (male and female) mice were initiated with 25 nmol DMBA and then treated with TPA (6.8 nmol) twice a week for the duration of the study. Mice were pre-treated with 340 nmol ACA or 2.2 nmol FA at 5 min prior to every TPA dose.

Another feature of the K5.Stat3C mice is the psoriatic phenotype. In the tumor study, mice exhibited multiple psoriatic plaques of varying degrees of severity (Figure 9). FA and ACA did not completely block this phenotype, but qualitatively appeared to modestly ameliorate the effect.

### ACA suppressed p65 phosphorylation in mouse skin

**Figure 10:**
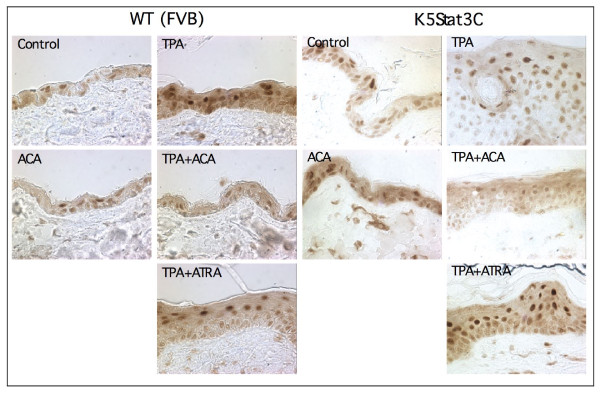
**Immunohistochemical staining of phospho-p65 NF-κB in mouse skin collected from the tumor study.** K5.Stat3C (male and female) mice were initiated with 25 nmol DMBA and then treated with TPA (6.8 nmol) twice a week for the duration of the study. Mice were pre-treated with 340 nmol ACA or 2.2 nmol FA at 5 min prior to every TPA dose.

An important consideration in the current study is whether ACA actually suppressed NF-κB activation in vivo in skin. Although it has previously been shown that ACA suppresses NF-κB activation, those studies were done in non-skin derived cultured cells [37,43]. Thus, to address whether ACA suppresses NF-κB activation in vivo in skin, sections of skin from K5.Stat3C and WT littermates (FVB background), treated with vehicle or TPA for 27 weeks, were stained immunohistochemically for the phospho-p65 NF-κB subunit. As shown in Figure 10, TPA increased the phosphorylation of the p65 NF-κB subunit (detected with an anti phospho-Ser529 p65 antibody) for both WT and K5.Stat3C mice, compared to control skin, indicating that TPA activates NF-κB signaling. ACA did not affect the level of phospho-p65 in control skin, but suppressed it almost to the control level in TPA treated skin. In contrast, ATRA did not suppress phospho-p65 levels. Use of primary antibody alone resulted in no staining (data not shown). We also note that phospho-p65 levels were higher in the K5.Stat3C skin for all treatment conditions except TPA + ACA, suggesting the possibility of cross-talk between Stat3 and NF-κB signaling in this system. Note also that the epidermal thickness was not increased by ACA or FA in the absence of TPA.

## Discussion

In 1976, Sporn defined chemoprevention as the use of specific natural or synthetic chemical agents to reverse, suppress or prevent the carcinogenic process to invasive cancer [44]. Due to the long latency period in human cancer development, effective but non-toxic agents should be used. Furthermore, studying the key cellular signaling pathways affected by known chemopreventive agents can be a logical starting point for gaining this understanding. The ultimate goal of such studies will be to prioritize the molecular targets and pathways that affect chemoprevention, such that other natural products that also impact these pathways can be exploited.

In the current study, one such molecular target was explored by using K5.Stat3C mice. These mice are exquisitely sensitive to TPA-induced skin tumor promotion [17], and also exhibit a psoriatic phenotype [11]. Originally we had hypothesized that ACA would be effective against TPA-induced skin tumor promotion in K5.Stat3C mice because it exhibits a range of chemopreventive activities. In the two-week TPA study, ACA was minimally effective, if at all. However, galanga extract containing equivalent amounts of ACA was highly effective at suppressing TPA-induced skin hyperproliferation and wet weight. The control, FA, was also very effective in these parameters, although it leads to tissue atrophy. This suggests that either additional components of the galanga extract are bioactive, or that the synthetic racemic ACA that is commercially available may be less effective than the pure *S*-enantiomer that is derived from the extract.

In the tumor study, both ACA and FA exhibited inhibitory effects against TPA-induced skin tumor promotion, although the subject size was not large enough to make solid conclusions with ACA. However, the purpose of the tumor study was to determine if ACA would be more effective under a tumor study protocol rather than the two-week protocol, and it appeared to be so. FA is a known inhibitor of epidermal DNA synthesis and suppresses tumor promotion [45] so it was expected to have an inhibitory effect. Furthermore, ACA strongly suppressed activated NF-κB in the skin of the K5.Stat3C mice from the tumor study. This is consistent with our previous report that orally administered ACA (100 mg/kg bw) inhibited lipopolysaccharide-induced NF-κB activation in the NF-κB-RE-luc (Oslo) luciferase reporter mice [46]. In a xenograft model, ACA (500 ppm) in combination with ATRA in the diet at 5, 10, and 30 ppm effectively suppressed human skin SCC SRB12-p9 tumor volume by 56%, 62%, and 98%, respectively [46].

**Figure 11:**
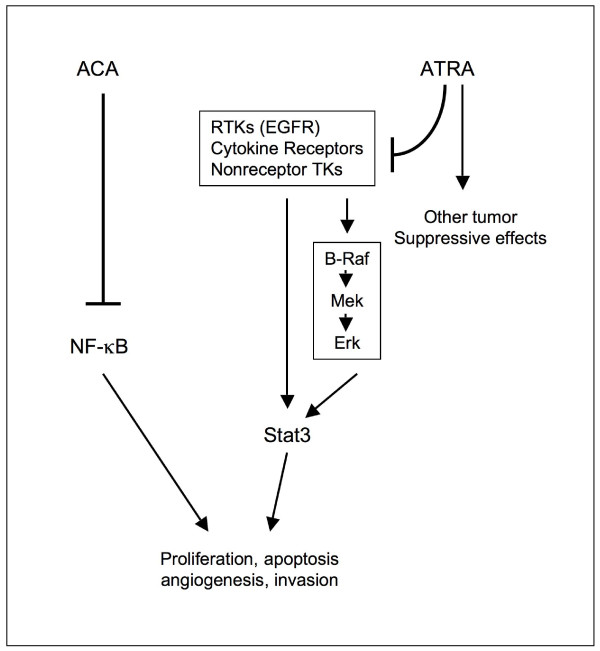
**Working diagram of the effects of ACA compared to ATRA in the NF-κB and Stat3 pathways, respectively.** RTK, receptor tyrosine kinase, TK, tyrosine kinase, EGFR, epidermal growth factor receptor.

In the K5.Stat3C study, all-trans retinoic acid (ATRA, 3.4 nmol) was also used as a potential inhibitor of TPA-induced skin tumor promotion [15]. ATRA is a well-known inhibitor of TPA-induced tumor promotion in SENCAR mice and the Clifford laboratory discovered that ATRA inhibits the B-Raf/Mek/Erk pathway [47] and suppresses the expression of p-Tyr^705^Stat3 [15]. In the K5.Stat3C mice, however, ATRA did not suppress the formation of carcinomas in situ or SCCs [15]. Since the mice express a constitutively active dimer form of Stat3 these results would suggest that ATRA suppresses events upstream of Stat3 activation. This explanation seems reasonable since B-Raf is upstream of Stat3. Taken together, these results are consistent with our previous cell culture findings that ACA was equally effective at blocking cell viability and/or proliferation in the 3PC mouse keratinocyte cell line vs. 3PC cells overexpressing Stat3C (Figure 1). Thus, it appears that both ACA and FA suppress events/pathways that are either downstream of Stat3, or are independent of Stat3. It should be noted that the FVB strain of mice used for generating the K5.Stat3C transgenic mice is not as sensitive to tumor induction in the 2-stage protocol as are SENCAR mice. This resulted in the lower total number of tumors per mouse observed for this experiment compared to a typical SENCAR experiment (data not shown). Also, the response of the K5.Stat3C mice to the DMBA/TPA protocol was not exactly as it was first reported [17]. This could be due to a number of factors, such as conducting the study in a different geographic region or differences in the breeding colonies. A working diagram is shown in Figure 11, in which ACA suppresses NF-κB activation, and ATRA inhibits the activation of Stat3.

## Conclusions

In conclusion, the current study reports, for the first time, that galanga extract effectively suppresses TPA-induced hyperproliferation, skin wet weight, and epidermal thickness in both WT and K5.Stat3C mice. Surprisingly, synthetic ACA only produced modest effects on these parameters. However, ACA strongly inhibited NF-κB activation in both WT and K5.Stat3C mice in the two-stage skin tumor study. ACA and FA also demonstrated a promising suppression of tumorigenesis in the K5.Stat3C mice, something that ATRA was not able to do. This may be useful clinically in individuals that already exhibit activated Stat3. These results further support the idea that targeting multiple pathways (Stat3, NF-κB) will be an effective strategy for chemoprevention.

## Competing interests

The authors report no conflicts of interest.

## Authors’ contributions

The study was overseen and directed by HKH and JLC. VB conducted the in vivo experiments, performed the statistics on the two-week in vivo studies, and wrote the original manuscript. ZS also contributed to the tumor study. JMM assisted in the revisions of the manuscript. RS conducted the statistical analyses on the cell culture study and the K5.Stat3C tumor study. JNG conducted the in vitro studies and assisted in the tumor study. MAC prepared and analyzed the galanga extracts. PA conducted the histopathological analyses. JD supplied the K5.Stat3C transgenic mice and assisted in the design and interpretation of the tumor study. All authors read and approved the final manuscript, which was revised by HKH.

## Grant Support

Grant from the Feist-Weiller Cancer Center, the Department of Pharmacology, Toxicology & Neuroscience. This research was also supported, in part, by National Cancer Institute grants 1K22CA102005-01A2 and 1R21CA149761-01A1(HKH). The content is solely the responsibility of the authors and does not necessarily represent the official views of the National Cancer Institute or the National Institutes of Health.
